# Accelerated Hypofractionated Radiotherapy in the Era of Concurrent Temozolomide Chemotherapy in Elderly Patients with Glioblastoma Multiforme

**DOI:** 10.7759/cureus.1388

**Published:** 2017-06-24

**Authors:** Liana Greer, Susan C. Pannullo, Andrew W Smith, Shoshana Taube, Menachem Z Yondorf, Bhupesh Parashar, Samuel Trichter, Lucy Nedialkova, Albert Sabbas, Paul Christos, A. Gabriella Wernicke

**Affiliations:** 1 Radiation Oncology, NewYork-Presbyterian/Weill Cornell Medical Center; 2 Neurological Surgery, NewYork-Presbyterian/Weill Cornell Medical Center; 3 School of Medicine and Dentistry, University of Rochester; 4 Division of Biostatistics and Epidemiology, Department of Healthcare Policy and Research, New York-Presbyterian/Weill Cornell Medical Center; 5 Stich Radiation Oncology, NewYork-Presbyterian/Weill Cornell Medical Center

**Keywords:** hypofractionated stereotactic radiotherapy, glioblastoma, elderly, radiation

## Abstract

Introduction

Patients with glioblastoma multiforme (GBM) over age 65 represent nearly half of those diagnosed per annum. They have a different tumor markers profile, physiologic reserve, and a median survival as low as three to four months. An optimal treatment strategy in older GBM patients remains undefined, with many patients receiving radiation in 30 treatments over six weeks, a regimen based on trials originally excluding patients over age 70. Recent studies have suggested reducing the number of treatments to 10-15 over two to three weeks with similar efficacy. We present an elderly population of patients treated with six radiation treatments.

Methods

After IRB approval, we reviewed the electronic medical records of 20 consecutive patients over the age 60 at diagnosis with GBM, treated with maximally safe neurosurgical resection, and adjuvant hypofractionated radiation (HFRT) and temozolomide (TMZ) between 2012 and 2015. HFRT was given every other weekday for two weeks, in a total of six fractions (6 × 6 Gy to contrast-enhancing tumor +5 mm and 6 × 4 Gy to fluid-attenuated inversion recovery (FLAIR) +2 cm) with concurrent TMZ (75 mg/m2 daily), followed by adjuvant TMZ (150-200 mg/m2 in 5/28 days). The response was assessed using the Macdonald and Revised Assessment in Neuro-Oncology (RANO) criteria, radiology reports, physician notes, and tumor board consensus notes. Descriptive statistics, overall survival (OS), progression-free survival (PFS), toxicity, and steroid use were calculated and compared to the historical controls of patients treated with a six-week radiation regimen of 60 Gy in 30 fractions with TMZ.

Results

The median age at diagnosis was 70.5 years (range: 61 - 82 years). Median pre-radiation Karnofsky performance scale (KPS) was 60 (range: 40 - 90). The median preoperative maximum gross tumor diameter on MRI was 3.6 cm (range: 1.8 - 6 cm). Six patients (30%) had a gross total resection (GTR), eight (40%) had a subtotal resection (STR), and six (30%) had biopsy only. The median progression-free survival was five months (95% (confidence interval) CI: 2.8, 16.4) and median OS of 14 months (95% CI: 5.0, upper limit not estimable). Of the 19 patients tested for isocitrate dehydrogenase-1 (IDH), 100% were negative. Of the eight patients who had MGMT methylation status results, four (50%) were positive for O^6^-methylguanine-DNA methyltransferase (MGMT) methylation. In the 18 patients who completed radiation, the HFRT treatment was well tolerated without any Grade 3/4 acute toxicities.

Conclusions

The accelerated adjuvant course of HFRT with TMZ used for the elderly with GBM decreases radiation treatment days to six. It was well tolerated in patients over 60 years of age and provided similar OS, PFS, minimal toxicity, and decreased steroid usage compared to historical controls treated with six or even two to three weeks of radiotherapy.

## Introduction

Glioblastoma multiforme (GBM) is the most common and the most aggressive malignant primary brain tumor in adults. An estimated 10,000 people are diagnosed with GBM in the United States each year [1], and despite aggressive treatment, the median survival is only 12 - 15 months from diagnosis [2]. Approximately half of these patients will be over the age of 65 at diagnosis [2], and these older patients have a different tumor marker profile and incidence [3], a different physiologic reserve, and a median survival as low as three to four months [4-5]. Given the proportion of GBM patients that are elderly and the especially poor prognosis in this subgroup of patients, it is imperative to have a well-tolerated treatment plan that balances best outcomes with quality of life. At the present time, however, the standard of treatment for the elderly GBM population is not clearly defined, and significant heterogeneity in the management of elderly patients with GBM is reported [6].

The current standard of care for GBM is based on a 2005 publication by Stupp, et al. and includes a maximum safe tumor resection, a standard course radiation therapy (RT) of 60 Gy in 30 daily fractions over six weeks, and concurrent and adjuvant temozolomide (TMZ) chemotherapy. This trial excluded patients over age 70, however, and the subgroup analysis found the survival benefit of this aggressive treatment strategy decreases in older patients [7]. With a median prognosis in the months, the quality of life considerations with standard RT (which requires six weeks of daily hospital visits) have also been raised, although the Association de Neuro-Oncologues d’Expression Francaise (ANOCEF) trial of patients over 70 years of age was stopped early after finding that post-surgery, RT compared to supportive care had a significant three month survival benefit without a difference patient performance or health-related quality of life [2]. In 2000, early success with TMZ and hypofractionated radiotherapy (HFRT) regimes prompted the Nordic trial in which GBM patients over the age of 60 were randomized to be treated with TMZ alone, HFRT to 34 Gy in 10 daily fractions alone, or standard RT. Standard RT was found to be inferior while the other two groups were not found to be significantly different from one another [2]. Similarly, in a prospective randomized control trial, Roa, et al. found hypofractionated RT of 40 Gy in 15 daily fractions over three weeks provided similar survival and palliative benefit to the standard six-week RT [8]. Additionally, Roa, et al. showed that fewer patients in the hypofractionated arm required corticosteroids and more completed RT [8]. These findings were further supported in the 2014 literature review by Arvold and Reardon, which concluded both HFRT and TMZ required fewer hospital visits, and HFRT versus RT has been shown to decrease required steroids [2]. Most recently, the 2017 randomized controlled trial by Perry, et al. specifically examined the role of TMZ in HFRT in patients over the age of 65 with GBM. In this study, 562 patients were randomized to receive HFRT of 40.05 Gy in 15 daily fractions with or without TMZ [9]. The median overall survival was statistically significantly longer in patients receiving TMZ [9], further supporting a regimen of HFRT with concurrent TMZ in elderly patients.

Based on the potential benefits of HFRT and TMZ, our institution has offered HFRT therapy of 6 Gy fractions in six fractions over two weeks, with concurrent and adjuvant TMZ, to selected patients as an alternate regimen to the standard course chemoradiation therapy of 60 Gy in 30 daily fractions over six weeks. This study reviews the results of HFRT with our cohort of elderly patients.

## Materials and methods

### Patient selection

Charts of consecutive patients with histologically confirmed GBM, over the age of 60 at diagnosis, and who were treated at a single institution by a single radiation oncologist specializing in central nervous system malignancies were reviewed for this study. Only patients who underwent maximally safe resection (or biopsy when considered inoperable), followed by HFRT and TMZ between 2012 and 2015 were included in this study.

The Weill Cornell Medicine Institutional Review Board approved this retrospective study and issued approval #0511008245.

### Surgery

All patients underwent a surgical procedure to histologically confirm the diagnosis of GBM. Maximally safe resection of the tumor was performed in all cases, except those in which the tumor was felt not to be resectable by the treating neurosurgeon. In the case of an unresectable tumor, a biopsy only was performed. All patients who underwent tumor resection had a postoperative MRI with contrast within 48 hours of the surgical procedure to determine the extent of resection (gross total resection (GTR), subtotal resection (STR) or biopsy only (Bx)).

### Adjuvant chemoradiation

Patients were offered the standard six-week course RT but declined and chose an alternative HFRT. The HFRT was given in six treatments every other day over two weeks. Radiation doses prescribed were 6 x 6 Gy to the contrast-enhancing tumor + 5 mm margin and 6 x 4 Gy to FLAIR hyperintensity + 2 cm margin. The dose painting intensity-modulated radiation therapy (IMRT) plans were generated from fused MRI and CT images. An additional 5 mm expansion was used to create planning target volumes (PTVs) for both the 6 x 6 Gy and 6 x 4 Gy doses. The brainstem, optic chiasm, and optic nerves were excluded from the final PTVs, if necessary, to keep the total radiation dose to the structure below Dmax 24 Gy (4 Gy/fraction) for the brainstem, and Dmax 21 Gy (3.5 Gy/fraction) for the optic chiasm and optic nerves. On each treatment day, patient positioning was verified, and adjusted if needed, by comparison of the cone beam CT images to the simulation CT. Concurrent TMZ (75 mg/m2 daily) was delivered during the two week HFRT period, and adjuvant TMZ (150-200 mg/m2 in five consecutive days, 28-day cycle) was delivered after the HFRT was complete.

### Data collection and statistics

Patient and disease characteristics, type of surgery, acute toxicity, dexamethasone usage, and follow-up outcomes were gathered through a review of the electronic medical records. Treatment response was assessed using the Macdonald and Response Assessment in Neuro-Oncology Criteria (RANO) criteria, the first available post-treatment MRI (at four weeks after HFRT) as well as subsequent radiology reports, physician notes, and available tumor board consensus notes. Time to recurrence was calculated as the interval between GTR and radiographic evidence of disease recurrence. Time to progression was calculated as the interval between biopsy or STR and radiographic evidence of disease progression. Follow-up time and overall survival were calculated from the date of the pathology confirmed diagnosis to the date of death or the date last confirmed alive. The incidence of toxicity was gathered from the medical records.

Progression-free survival (PFS), defined as either the time from to first documented disease progression or recurrence, was calculated for each patient. Patient outcomes were classified as no evidence of disease (NED), alive with disease (AWD), dead of disease (DOD), or dead of other causes (DOC). DOC was assigned in the case that a patient died with no clinical or radiographic signs of disease recurrence or progression since the most recent follow-up visit. Overall survival (OS) was calculated with the additional use of the Social Security death index data when available. The Kaplan Meier product-limit method was used to estimate PFS and OS in the entire cohort. Patients who did not experience progression, death, or were lost to follow-up by the end of the study period were censored in the analysis.

## Results

Between 2012 and 2015, 35 GBM patients were treated with accelerated hypofractionated RT. Of these, 15 patients were excluded from analysis because they were under 60 years of age at the time of diagnosis. The remaining 20 patients, who were over age 60 at diagnosis, were included in the analysis. Patient gender, age, surgery type, tumor location, size, tumor pathology, and pretreatment KPS are summarized in Table 1.

**Table 1:** Patient and Tumor Characteristics KPS: Karnofsky performance status; Bx: biopsy only; STR: subtotal resection; GTR: gross total resection; MGMT: O-methylguanine-DNA-methyltransferase; IDH: isocitrate dehydrogenase-1

Patient	Gender	Age at diagnosis	Surgery type	Side	Location	Size	MGMT methylation	IDH	Pre-RT KPS
1	Male	61	GTR	L	Temporal	2.6	unknown	neg	80
2	Female	66	GTR	L	Parietal	2.5	no	neg	90
3	Male	78	GTR	L	Parieto-occipital	4.8	unknown	neg	60
4	Male	82	GTR	R	Frontal	1.9	unknown	neg	70
5	Female	62	GTR	L	Parietal	4.5	yes	neg	80
6	Female	64	GTR	R	Frontal	2.7	no	neg	60
7	Male	61	STR	L	Frontal	2.5	yes	neg	70
8	Male	69	STR	R	Occipital	5.8	yes	neg	60
9	Female	69	STR	R	Thalamus	3.2	unknown	unknown	60
10	Male	70	STR	L	Temporal and hypothalamus	3.5	no	neg	60
11	Male	76	STR	L	Frontal	4	unknown	neg	70
12	Male	76	STR	R	Parietal	3.7	no	neg	40
13	Female	81	STR	R	Frontal	5.7	unknown	neg	50
14	Male	74	STR	R	Frontal	1.8	unknown	neg	40
15	Female	61	Bx	L	Thalamus	6	unknown	neg	60
16	Female	71	Bx	L	Parietal	2.9	unknown	neg	50
17	Female	71	Bx	L	Parietal	4.9	unknown	neg	50
18	Male	76	Bx	L	Frontal	2.5	unknown	neg	40
19	Male	72	Bx	L	Temporal and insular	5.3	yes	neg	60
20	Male	65	Bx	R	Parietal	3.6	unknown	neg	40

The median age of diagnosis was 70.5 years (range: 61 - 82 years). Median pre-radiation KPS was 60 (range: 40 - 90). The median preop maximum dimensions of the gross tumor on MRI was 3.6 cm (range: 1.8 - 6 cm). Six (30%) patients had GTR, eight (40%) had STR, and six (30%) had Bx only. Of the 19 patients tested for IDH, all were negative. Of the eight patients who had MGMT methylation status results, four (50%) were positive for MGMT methylation.

Four patients did not complete the treatment. Patient 5 completed the pre-RT simulation, but the surgical wound developed an infection, HFRT was put on hold, and she passed away from complications of the infection. Patient 14 took a one-week break during HFRT and was subsequently lost to follow-up. Patient 19 completed pre-RT simulation but was then lost to follow-up without beginning the RT. Patient 20 received only two fractions of HFRT before being lost to follow-up.

**Figure 1:**
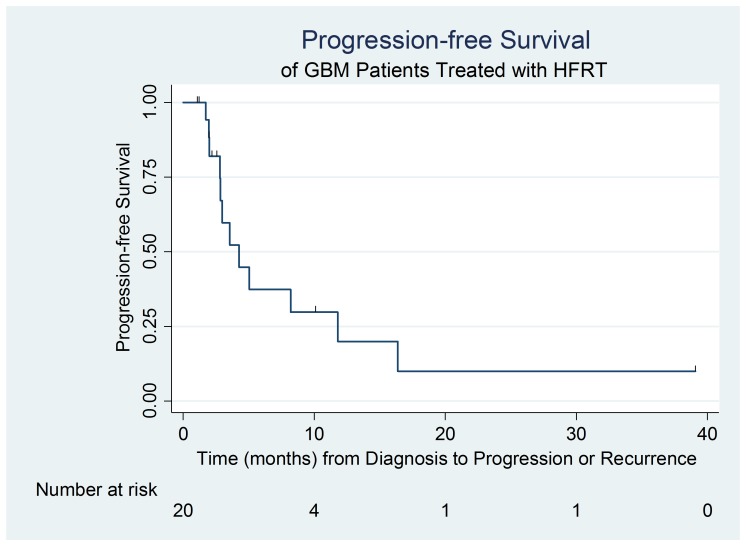
Progression-free survival outcomes of GBM patients treated with HFRT and TMZ GBM: glioblastoma multiforme; HFRT: hypofractionated radiation; TMZ: temozolomide

**Figure 2:**
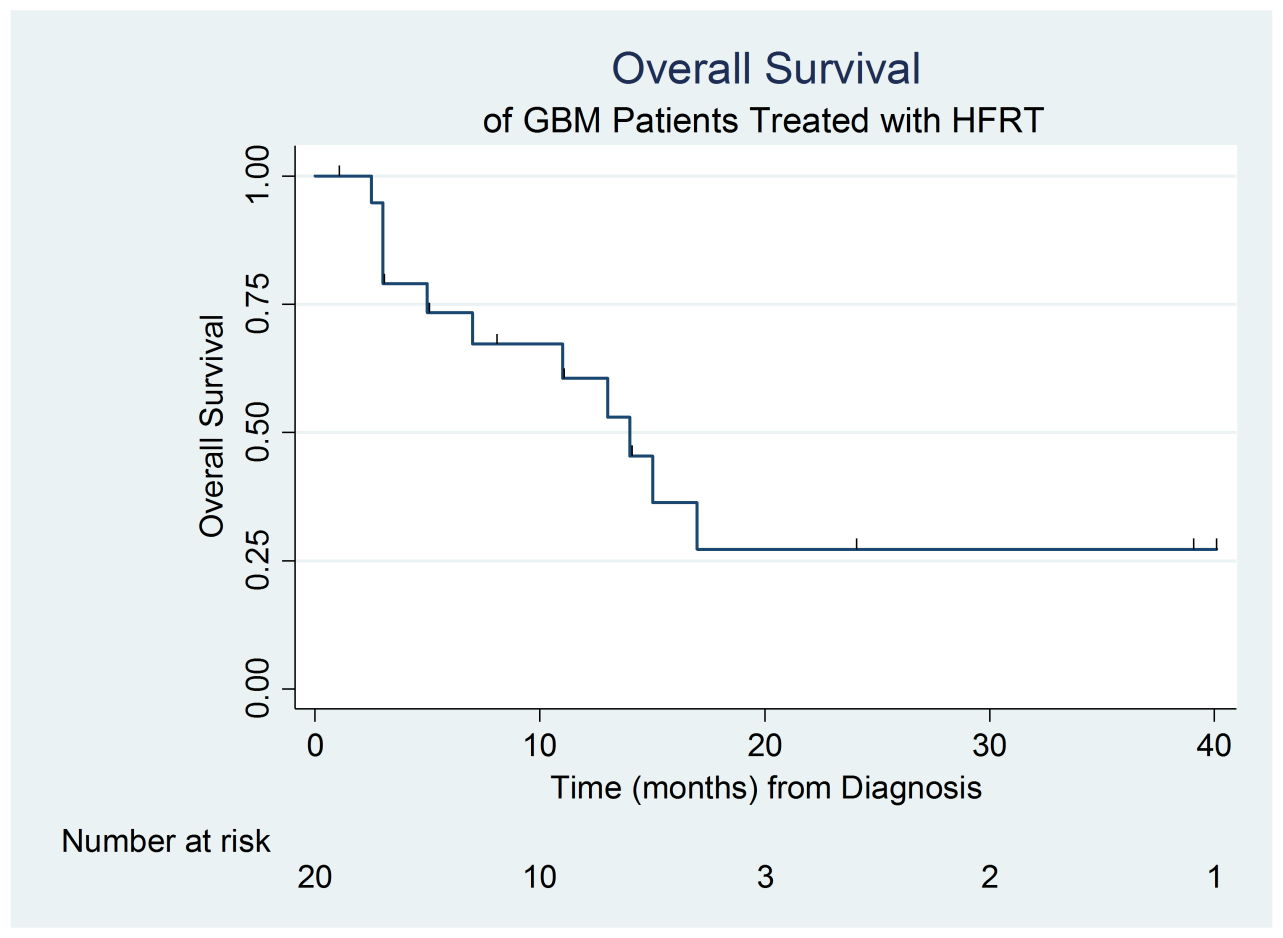
Overall survival outcomes of GBM patients treated with HFRT and TMZ GBM: glioblastoma multiforme; HFRT: hypofractionated radiation; TMZ: temozolomide

During the study period, examining all 20 patients included in the study group, 12 (60%) patients developed recurrence or progression and eight (40%) confirmed deaths occurred. The median follow-up time to progression was three months (range: 1 - 39), and the median follow-up time to death was 9.5 months (range: 1 - 40 months). The median PFS was 4.3 months (95% confidence interval (CI): 2.8, 11.8). Median survival was 12 months (range: 3 - 40 months), excluding the four patients lost to follow-up, or 10 months (range: 1 - 40 months) if including all patients using last record of contact as last date alive. Median OS was 14 months (95% CI: 5.0, upper limit not estimable). A survival analysis was again performed, excluding two patients who entered the study but did not initiate the HFRT treatment. In this subset sample, median PFS was five months (95% CI: 2.8, 16.4) and median OS was 15 months (95% CI: 7.0, upper limit not estimable). Figure 1 depicts the progression-free survival outcomes of patients in the study group. Figure 2 depicts the overall survival outcomes of patients in the study group.

In the first post-RT scans, eight patients had stable disease, and eight were found to have progression. Two patients were lost to follow-up before the first post-RT scan. Four of the patients with stable disease eventually developed progression. Three patients with disease progression were still alive at follow-ups of 14, 24, and 40 months. Two patients with no radiographic evidence of disease were alive at follow-ups of 11 and 39 months. Time to recurrence or progression, overall survival, follow-up, toxicity, treatment response, and status are summarized in Table 2. 

**Table 2:** Outcomes and Toxicity by Patient Some patients were lost to follow-up. For these patients, ^a^ denotes patients lost to follow-up after the number of months indicated, and ^b^ denotes patients lost to follow-up without subsequent imaging. N/A: not available; AWD: alive with disease; DOD: dead of disease; MRI: magnetic resonance imaging; NED: no evidence of disease; OS: overall survival; RT: radiation therapy; RTOG: Radiation Therapy Oncology Group; TX: treatment

Patient	Months to recurrence or progression	OS (months)	Follow-up (months)	RTOG grade 3/4 toxicity	Response at first post-Tx MRI	Status
1	5	13	13	None	Stable	DOD
2	4	alive at 14	14	None	Progression	AWD
3	8	8^a^	8	None	Stable	Lost to follow-up
4	None at 2	5	5	None	Stable	DOC
5	2	3	3	N/A didn't receive RT	Progression	DOD
6	None at 11	alive at 11	11	None	Stable	NED
7	11	alive at 39	39	None	Stable	NED
8	12	alive at 40	40	None	Stable	AWD
9	4	15	15	None	Progression	DOD
10	16	17	17	None	Progression	DOD
11	3	alive at 24	24	None	Progression	AWD
12	None at 1	5^a^	5	None	Stable	Lost to follow-up
13	2	3	3	None	Progression	DOD
14	N/A^a^	14	14	None	N/A^b^	DOD
15	2	7	7	None	Progression	DOD
16	3	3	3	None	Stable	DOD
17	3	11	11	None	Progression	DOD
18	N/A^a^	1^a^	1	None	N/A^b^	Lost to follow-up
19	N/A^a^	2^a^	2	N/A didn't receive RT	N/A^b^	DOD
20	None at 1	3^a^	3	None	Stable	Lost to follow-up

In the 18 patients who completed radiation, the HFRT treatment was well tolerated without any Radiation Therapy Oncology Group (RTOG) Grade 3/4 acute toxicities. Among lower grade toxicities, fatigue was the most commonly noted effect.

Of the 18 patients who received radiation, 15 (83%) were taking dexamethasone at the start of RT with a median dose of 4 mg/day (range: 0 - 24mg/day). Two of the three not taking dexamethasone remained without dexamethasone, while one started on low dose 4 mg/day dexamethasone after the first week of RT due to headaches. All 15 patients taking dexamethasone did not require an increase in dosage during treatment, and 6/15 (40%) were able to taper their dosage during or soon after RT.

## Discussion

The standard of care for older GBM patients is currently controversial. Although treatment for GBM patients has evolved, many of the trials leading to today’s standard of care initially excluded patients in their 60's and 70's [10]. Today’s standard of care remains maximum safe tumor resection, standard course RT of 60 Gy in 30 daily fractions over six weeks, and concurrent and adjuvant TMZ chemotherapy. Support of this regimen originated with the 2005 publication by Stupp, et al., which studied 573 patients aged 18 - 70 with a median age of 56 [11]. The authors found RT with TMZ chemotherapy was better than RT alone, leading to the establishment of TMZ chemotherapy for GBM and today’s standard of care. Subgroup analyses of older patients in this group, though limited by size, noted a decreasing of benefits among older adults.

It is important to specifically examine treatment in older GBM patients because treatment results from younger GBM patients may not generalize to older GBM patients. It has been recognized that prognosis is significantly poorer in elderly GBM patients than younger patients for some time, with advanced age an adverse, independent prognostic factor [2, 10]. Some studies quote the median survival after diagnosis as low as three to four months [4-5]. This may be due to less aggressive treatment in elderly patients, but it may also demonstrate a different physiologic reserve and tumor characteristics. For example, Eckel-Passow, et al. showed older patients have tumor marker incidence significantly different than younger patients, which impacts survival. For example, IDH-1 is prognostically favorable among gliomas but is almost entirely absent from elderly GBM [2-3]. MGMT methylation, present in 40 - 60% of GBM tumors in the elderly, is a positive prognostic marker. In the Randomized Phase III Study of Sequential Radiochemotherapy of Anaplastic Glioma with PCV or Temozolomide (NOA-04) and the Neuro-Ophthalmology Research Disease Investigator Consortium (NORDIC) trials, patients with MGMT methylation survived approximately three months longer than their peers regardless of treatment modality [2]. Arvold and Reardon additionally determined that many tumor markers correspond with variable prognoses based on patient age [2]. For example, TP53 was found to predict reduced survival in patients over 70, while in patients under 70, it was associated with improved survival. Tumor markers in our cohort of patients reflect the expected tumor marker incidence in older adults. None of the patients had the prognostically favorable IDH-1 mutation. Of patients tested for MGMT methylation, 50% (four patients) were positive and 50% were negative.

As differences in characteristics of older GBM patients emerged, several studies were developed to examine treatment methodologies for GBM specifically in older patients. Roa, et al. conducted a prospective randomized control trial with GBM patients over 60 years of age. Patients were treated with either the standard six-week RT or hypofractionated RT of 40 Gy in 15 daily fractions over three weeks. Both groups had similar survival and palliative benefits; fewer patients in the hypofractionated group required corticosteroids, and a greater number of patients in the hypofractionated group were able to complete RT [8]. In another study of older adults, the prospective randomized NORDIC trial enrolled GBM patients over the age of 60 to be treated with TMZ alone, HFRT to 34 Gy in 10 daily fractions alone, or standard RT. Standard RT was found to be inferior, while the other two groups were not found significantly different from one another [2].

With growing evidence of the benefits of TMZ in GBM and evidence that HFRT was non-inferior to standard RT with added benefits of lesser steroid usage and fewer treatment days, our institution was motivated to offer HFRT therapy of 6 Gy fractions over two weeks with concurrent and adjuvant TMZ to selected elderly patients as an alternate regimen to the standard six-week course of chemoradiation therapy or the two to three-week course offered to the elderly. Hypofractionation to 6 Gy was selected based on institutional experience and the promising results obtained utilizing 6 Gy fractions in the Phase II trial reported by Reddy, et al. [12]. Further support for this regimen was based on the absence of increased toxicity found in HFRT trials and the potential synergistic effects of sensitizing tumor cells with TMZ while utilizing accelerated HFRT, which increases tumor cell death with increased dose per fraction and reduces the opportunity for tumor repopulation with reduced overall treatment time [13].

The results of our study show a median PFS of 4.3 months (95% CI: 2.8, 11.8) and median OS of 14 months (95% CI: 5.0, upper limit not estimable). Other studies of GBM patients over the age of 60, utilizing RT alone, TMZ alone, or RT and TMZ, have found a median OS from four to 15 months [14]. Of these, studies that treated patients with both RT and TMZ reported a median OS ranging from nine to 15 months, with radiation doses and fractionation of 60 Gy/30, 59.44 Gy/33, and 40 Gy/15 [14]. Our results compare favorably with these reported survival data, and despite our increased dose per fraction with a radiation dose and fractionation of 36 Gy/6, 93% of patients in our study taking dexamethasone at the start of HFRT had their dose remain stable or decreased during treatment. Three other patients were not on dexamethasone when HFRT started and two completed the course without requiring any corticosteroids. This suggests that hypofractionated RT may be better tolerated by patients, in line with what other studies have found [8, 15]. We also found no RTOG Grade 3/4 side effects from the treatment.

Considering our patients compare favorably on OS and dexamethasone use, we suggest hypofractionated RT to 36 Gy in 6 Gy doses may be a viable treatment option for older patients which needs to be tested in a prospective arena. Our patients compared favorably to historical controls despite a potential selection bias for frailer patients [14]. Since our HFRT was offered only to selected patients and as an alternative to the standard treatment, the benefits in OS and decreased steroid use may be understated. Additionally, with this HFRT regimen, patients visit the hospital three times a week for two weeks, for a total of six RT sessions. This is a significantly less burden of visits than the standard regime, which requires daily visits for six weeks, for a total of 30 RT sessions, or even 10 - 15 sessions. While we did not conduct a quality of life questionnaire, based on the results of other studies, this may be a positive result for patients and caregivers, especially in the setting of a disease with a median life expectancy measured in months [15-16]. Additionally, HFRT may allow better resource utilization and decreased wait times for patients due to the fewer number of RT treatment days, an important consideration as the incidence of GBM has been increasing over the past several decades in a trend that is expected to continue [11]. 

Two recent studies have examined tolerability and benefits of HFRT with TMZ over a time course closer to our regimen than previous literature studies. Roa, et al. conducted a randomized phase III study comparing TMZ with RT to 25 Gy/5 over one week to TMZ with RT to 40 Gy/15 in three weeks and found the short-course RT to be non-inferior [15]. Omuro, et al. treated patients with TMZ and RT of 36 Gy/6 over two weeks with the addition of bevacizumab [16]. Although these studies were not restricted to older adults, their findings of safety and promising OS with a median OS of 7.9 months and 19 months, respectively, reported, are similar to what we have found in our treatment with HFRT. A randomized Phase II trial of HFRT or proton beam therapy versus traditional RT with concomitant and adjuvant TMZ is currently enrolling patients, which, while it does not focus exclusively on older patients, may provide additional guidance. We hope in the future to improve our understanding of the role of tumor markers, HFRT, and TMZ in older patients with GBM and develop improved therapies to extend survival and quality of life for all patients with this debilitating disease

Although we have seen favorable results that correspond with those which were predicted based on the literature, this study had several limitations. It was a small cohort, at a single institution, and had a bias of a retrospective study. Due to relatively small number of death events as well as right-censoring, the 95% confidence limits were not entirely estimable. As a relatively rare disease, the small cohort was accepted so that the initial analysis could begin within a few years of the start of the HFRT. A control arm was not identified for this project due to the fact that the treatment was only offered to selected patients, and only some of these, possibly biased for worse prognosis, consented. For this new treatment, the authors felt historical controls with overall survival data from many centers could give the best insight into the favorability of HFRT. Our small cohort and potentially biased selection based on a single institution, however, does call into question whether these results would generalize equivalently to all older GBM patients.

## Conclusions

As approximately half of GBM patients are over the age of 65 at diagnosis, it remains a critical goal to develop a standard of care treatment for patients in this population that balances best outcomes with quality of life. Recent reports focusing on an older population, including this one, have demonstrated promising results for HFRT with TMZ chemotherapy. Our study has found HFRT with TMZ to be well tolerated while providing similar OS and decreased steroids compared to historical controls. To fully validate our results in support of HFRT and TMZ, a prospective trial is needed. 
